# The Trade-Off between Female Fertility and Longevity during the Epidemiological Transition in the Netherlands

**DOI:** 10.1371/journal.pone.0144353

**Published:** 2015-12-17

**Authors:** Ralf Kaptijn, Fleur Thomese, Aart C. Liefbroer, Frans Van Poppel, David Van Bodegom, Rudi G. J. Westendorp

**Affiliations:** 1 VU University Amsterdam, Amsterdam, The Netherlands; 2 Netherlands Interdisciplinary Demographic Institute, The Hague, The Netherlands; 3 University Medical Centre Groningen, Groningen, The Netherlands; 4 Leyden Academy for Vitality and Aging, Leiden, Netherlands; 5 University of Copenhagen, Copenhagen, Denmark; London School of Hygiene and Tropical Medicine, UNITED KINGDOM

## Abstract

Several hypotheses have been put forward to explain the relationship between women’s fertility and their post-reproductive longevity. In this study, we focus on the disposable soma theory, which posits that a negative relationship between women’s fertility and longevity can be understood as an evolutionary trade-off between reproduction and survival. We examine the relationship between fertility and longevity during the epidemiological transition in the Netherlands. This period of rapid decline in mortality from infectious diseases offers a good opportunity to study the relationship between fertility and longevity, using registry data from 6,359 women born in The Netherlands between 1850 and 1910. We hypothesize that an initially negative relationship between women’s fertility and their longevity gradually turns less negative during the epidemiological transition, because of decreasing costs of higher parities. An initially inversed U-shaped association between fertility and longevity changes to zero during the epidemiological transition. This does suggest a diminishing environmental pressure on fertility. However, we find no evidence of an initial linear trade-off between fertility and post-reproductive survival.

## Introduction

Although the relationship between women’s fertility and their post-reproductive longevity has been extensively studied, the nature of this relationship remains unclear. A meta-analysis of 31 studies on this topic did not show a consistent pattern. The relationship between women’s fertility and longevity can be negative, positive, or absent [1, 2]. In line with these equivocal results, several hypotheses have been put forward in order to explain these results. Positive effects of fertility on longevity have been explained by the health benefits that older women may gain from the social and material support that their children provide to them [1, 3, 4]. Another explanation for a positive relationship between women’s fertility and longevity is the healthy mother effect: women who are healthy may both have a high number of children and live long [1, 5, 6].

A negative relationship between fertility and longevity can be understood as an evolutionary trade-off between reproduction and body maintenance. The disposable soma theory [7] emphasizes energetic and metabolic costs associated with reproduction. This cost of fertility could consist of maternal depletion: bearing and raising a large number of children is physically demanding and may have deteriorating effects on a woman’s health and life span [8–10]. Maternal depletion may be especially important if a woman becomes widowed while she has to care for her young children [11]. More specifically, such a trade-off can also be explained by the theory of antagonistic pleiotropy, where genes that increase reproductive potential early in life increase risk of disease and mortality later in life [12]. As an example, but not exclusively, the functioning of the immune system could explain such a trade-off. In line with theory, women with a genetic profile that makes them more fecund have been found to be more susceptible to die from infectious diseases [13–15]. Moreover relatives of less-fecund and long-living individuals have also been found to have lower mortality [16] whereas offspring of fecund mothers are found to be more susceptible to mortality in an environment with high infectious mortality [17] Genetic studies have pointed to contributing loci [12] but not a single genetic indicator of this association [18].

The diverse outcomes of phenotypic studies could be explained by diversity in the populations studied and by insufficient controlling for environmental factors [16, 19]. Differences in environmental pressures [20] and in resources of the parents [21] can lead to differences in selection on fertility. Some even suggest that the trade-off may only be visible when women have limited resources [22]. In line with this assumption negative association between fertility and mortality are more prevalent in historic and natural fertility populations. In contemporary populations, a U-shaped curve seems more common: women with low and high parities die earlier than women with parity in between. The lowest mortality was found in mothers of one to four children [1, 23] and in mothers of two to four children [1, 24]. This difference between historic and contemporary populations may be attributable to methodological differences, such as more reliable data on nulliparous women in recent cohorts. A recent review study focusing on more reliable data concludes that also in historic populations low, but not null parities often correlate with long post-reproductive health [2]. The finding of a U-shaped association in more recent populations could also mean that the environmental pressures on (low) parity have changed [1]. Unhealthy women can reproduce more often under more prosperous conditions like those in contemporary populations, but parity may be lower than that of healthy women. As a consequence, both the nulliparous women and the women with low parities might represent unhealthier categories than in historical and natural fertility populations, causing higher mortality. Unobserved health factors and differences in socioeconomic conditions could suppress or confound an effect of fertility on mortality.

In this study, we explore evidence for an evolutionary trade-off by investigating the relationship between fertility and post-reproductive longevity during the epidemiological transition in the Netherlands. This period is marked by a sharp decline in mortality from infectious diseases and greater prosperity. This change entails a lessening of environmental pressure on fertility and mortality which could affect the trade-off between fertility and mortality. Following the theoretical framework of the disposable soma theory, we expect an initially negative association between fertility and post-reproductive survival to become weaker with decreasing environmental hazard.

**Fig 1:**
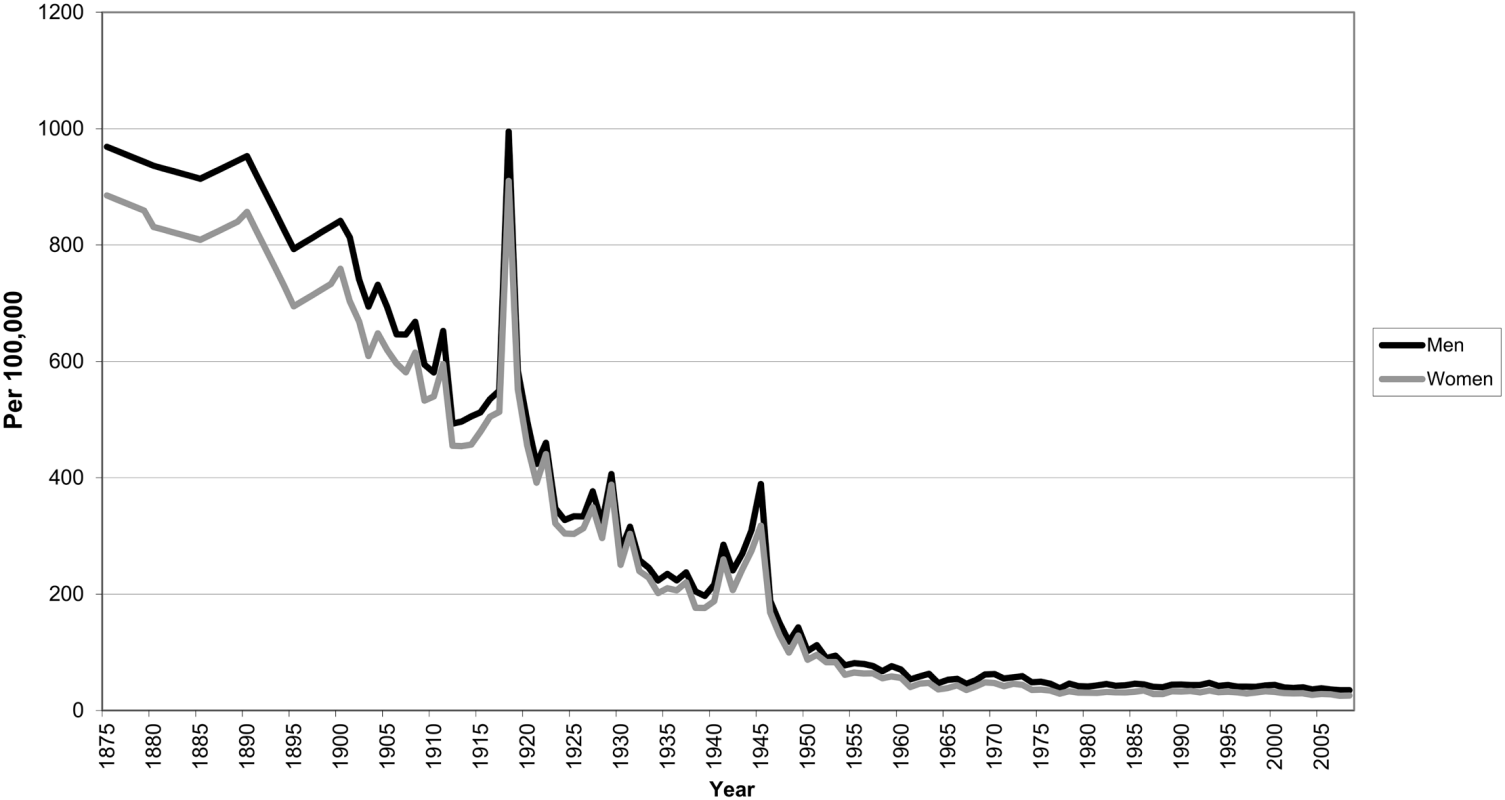
Age-standardized mortality from infectious diseases for men and women in the Netherlands. Source: Statline Database Statistics Netherlands (statline.cbs.nl).

We take mortality from infectious diseases as a marker of environmental hazard. As a result of improved wealth, sanitation and medicine, mortality from infectious diseases sharply declined in the nineteenth and twentieth century all over Europe [13, 14, 25]. In the Netherlands, female age-standardized mortality from infectious diseases dropped from 885 deaths per 100,000 in 1875 to 26 deaths per 100,000 in 2008. For men, the age-standardized mortality from infectious diseases showed an even stronger decline [26]. This decline in mortality from infectious diseases is shown in Fig 1. The peak in mortality around 1918 is mainly caused by the Spanish flue pandemic [27]. Infectious diseases were the main cause of mortality in pre-modern times [13, 14, 25], but by 1945 in the Netherlands this role has been taken over by cardiovascular diseases [26]. This means that mortality has become less conditional upon adverse environmental conditions.

Together with the drop in mortality from infectious diseases, total fertility rates in the Netherlands dropped from about 5.4 in 1875 to 1.8 in 2008 [28]. Environmental and health risks are a smaller constraint on the number of children a woman gets because fertility is already limited for social reasons such as a smaller desired family size. This desired family size is also in the reach of many women with weaker health.

Taken together the diminishing impact of environmental hazards on survival during the epidemiological transition and the diminishing impact of health on women’s fertility in the same period suggest that the evolutionary trade-off between fertility and survival will have diminished during the epidemiological transition. We expect a negative relationship between women’s fertility and longevity before the start of this transition. When the epidemiological transition progresses this relationship will gradually turn weaker. In this study, we test this hypothesis using demographic data from the nineteenth and twentieth century in the Netherlands. Our dataset includes information on the life courses of women and men. Given that our theoretical expectations only apply to women, and given that fertility data of men may suffer from underreporting [29], we focus our analyses on women only.

## Methods

### Data

**Fig 2:**
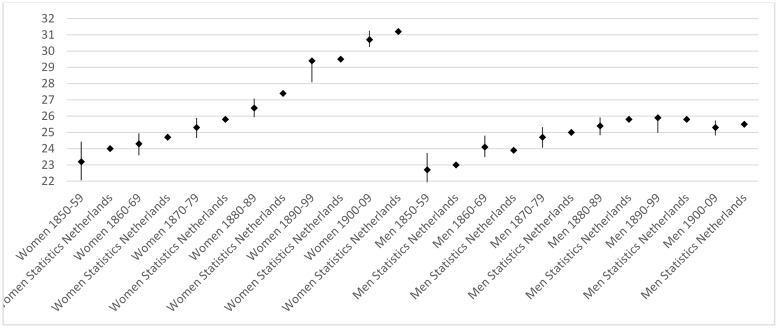
Trends in life expectancy at age 50, by gender and age cohort, among the HSN sample and the population (for HSN mean, lower and upper confidence interval). Sources: HSN (own calculation), and Statline Database Statistics Netherlands (statline.cbs.nl).

The data for this study come from the Historical Sample of the Netherlands (HSN). The HSN is an on-going data collection programme that eventually will contain the life courses of a representative sample of about 77,000 people born in the Netherlands during the period 1812–1922. In this study, we use the 2013.01 release of the HSN that contains information on the life courses of individuals born between 1850 and 1909. We excluded cohorts born from 1910 onwards, as not all sample members in these cohorts had died in 2013. Information on births produced by these cohorts comes from records that are only made available after the death of the individuals concerned. Because this information is not available for censored individuals, this could bias the results. Life-course information is collected from several Dutch administrative sources. These sources include birth, marriage and death certificates, population registers, personal cards and the municipal basic administration. The focus of this article is on post-reproductive longevity. To examine the quality of our data, we calculated the life expectancy at age 50 for men and women per 10-year birth cohort in the HSN and compared these to those for the total population as published by Statistics Netherlands. As can be seen from Fig 2, differences between our sample and the population data are only minor. In addition, the trends in life expectancy at age 50 across cohorts in our sample mirror those in the population. Among women, a steady increase is visible between cohorts 1850–59 and 1900–09. Among men, an initial increase in life expectancy at age 50 is followed by stability for cohorts born from 1880 onwards.

Because of our focus on post-reproductive longevity, we only included individuals who were ever married and survived at least to age 50. We also only included individuals who had their last child before age 50, so that our sample only contained individuals who were truly in their post-reproductive life phase. In the sample of individuals who were ever married and reached age 50, 0.2% of the women and 3.7% of the men had their last child when they were 50 or older. Our final sample consisted of 6,359 women and 6,480 men.

### Measures

#### Dependent variable

Our dependent variable is post-reproductive lifespan. Post-reproductive lifespan is measured as the age at death minus 50 years. The median post-reproductive lifespan among the ever-married is 27.6 years for women and 25.2 years for men. For 0.9% of the women and 0.8% of the men, post-reproductive lifespan is censored at the time of their last appearance in the registry system, as no information on their time of death is available.

#### Independent variables

Our main independent variable is the number of children an individual ever had. This information is gathered from the municipal registers and from the personal cards. The median number of children born to ever-married women is 3.96.

We control for a number of possible confounding factors. First, we control for year of birth, because both post-reproductive lifespan and fertility correlate with calendar time. In addition, to test if the relationship between fertility and post-reproductive lifespan changed during the epidemiological transition, the number of children that an individual ever had is interacted with the year of her birth. Because the range in birth years is quite large–which would result in very small regression coefficients–we divide the original scale by 10. Socio-economic status can correlate with fertility and post-reproductive lifespan as well. For this reason we also control for socio-economic status. Our measure of socio-economic status focuses on social power, which is defined as the potential to influence one’s life chances through the control of economic and cultural resources [30]. It consists of five classes–running from lower skilled workers to middle/upper class, and is treated as a continuous measure in our analyses. For women, the measure of socio-economic status is based on the last reported occupation of their husband, or–if that is missing–on their own last reported occupation. For men, the measure of socio-economic status is based on the last reported occupation of themselves, or–if that is missing–on their wives’ last reported occupation.

We also control for factors related to the support of a spouse and the opportunity to get children. These factors are: the number of reproductive years (age 15 to 50) that the individual was married and the age difference between husband and wife (for multiple marriages: age difference in the last marriage). The number of reproductive years that the individual was married is the sum over all marriages, irrespective of any children born within the marriage. 7.2% of the women and 10.8% of the men in our sample married more than once. The age of the spouse is missing for 12.6% of the women and 10.9% of the men in our sample. We impute the mean age difference for these cases. Because we expect the number of children to have a negative effect on women’s post-reproductive lifespan, we also control for widowhood. Widowhood is included as a time-varying covariate in the analyses. The median age at widowhood is 75 years for women and 86 years for men. 56.7% of the women and 28.0% of the men in our sample experienced widowhood. Finally, we included the fraction of children who died before the age of 5 as a general indicator of health. Gagnon et al. [31] argue that a high fraction of children dying could signify poor childrearing practices or living in a bad environmental setting. Given the skewness of this variable, we constructed a dummy variable indicating whether or not women have experienced the death of one or more of their children before the age of five. This is experienced by 28.5% of all ever-married women who had children.

In 1994, the Dutch population registration system changed from a system with personal cards to a system of digitalized record keeping. The information on the personal cards was in some cases not completely copied into the new system. Underreporting of the number of children of women may occur if their husband died after 1994. For this reason, we add a dummy that indicates the death of the husband after 1994 to our analyses of the women in our sample. The descriptive statistics of all variables are shown in Table 1.

**Table 1:** Descriptive statistics of independent variables for ever-married women (N = 6,359).

	Mean^a^	SD^b^
Year of birth	1886	14.65
Social power	2.78	1.28
# reproductive years married	22.98	6.49
Age difference husband and wife	2.28	4.45
Age at widowhood	65.46	13.24
Husband died after 1994	0.9%	
# children	3.96	3.37

^a^ Percentage shown if variable is dichotomous.

^b^ Not shown if variable is dichotomous

### Statistical analysis

To get a better grasp of overall mortality differences by parity in our sample, we first estimate Kaplan-Meier survival curves by parity. Next, we test our hypotheses formally, using a Cox regression model [32]. In this model we estimate the effect of a set of time-constant and time-varying covariates on the mortality rate after age 50. We analyze two models. Model 1 includes the number of children and the control variables. In this model, we test our first hypothesis that there is a trade-off between the number of children and post-reproductive longevity for all birth cohorts from 1850 to 1909 together. Model 2 includes the same variables as Model 1 plus the interaction between the number of children and the year of birth. In this model, we test our second hypothesis that the trade-off between the number of children and post-reproductive longevity diminishes during the epidemiological transition. All explanatory variables are centered at their means. We test whether the effects of parity are proportional.

## Results

**Fig 3:**
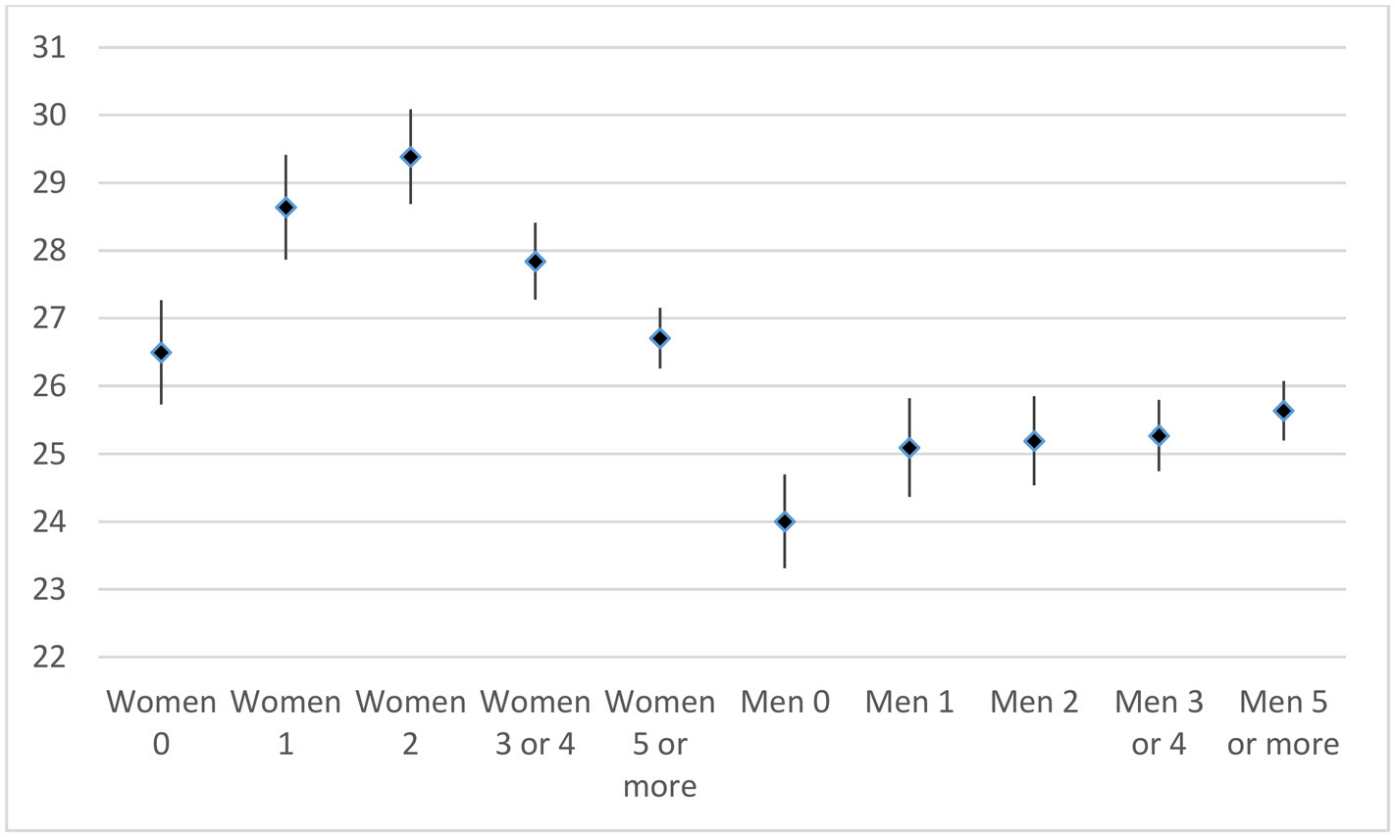
Mean survival time (in years) after age 50, for ever-married women and men, by parity (mean, lower and upper confidence interval). Note. The number of cases varied between 752 and 2246 per category.

Fig 3 presents mean survival times in years after age 50 for ever-married women and men with different parities, averaged for the birth cohorts between 1850 and 1909. Mean survival times are lowest for nulliparous women (26.5 years) and for women with five or more children (26.7 years). They are highest for women with two children (29.4 years). Overall, the estimates show a curvilinear relationship with survival times, increasing up to two children and decreasing at higher parities. All estimates differ at p < .05, except those between 0 and 3 or 4 children and between 1 and 5 children or more. Among men, the differences are much less pronounced. Men without children have the lowest mean survival time (24.0 years) and those with 5 children or more have the highest mean survival (25.6 years). Among men with children, mean survival times are not statistically significant different from each other.

Given that our hypotheses focus on women rather than men, we will restrict our multivariate analyses to women only. In addition, given that our focus is on women who bore children, we only focus on women who bore at least one child. The results of the Cox regression analyses are shown in Table 2. To ease the interpretation of the regression coefficients, we present the exponentials of the coefficients which can be interpreted as hazard ratios. The table also shows the confidence intervals for the hazard ratios. In Model 1, we present the results of a model without interactions between number of children and birth cohort. There is no statistically significant effect for occupational class, although there is a tendency (p = .095), for the post-reproduction mortality rate to decrease with occupational class standing. The number of reproductive years in marriage and the experience of widowhood have no effect on post-reproductive mortality. However, the larger the age differences between spouses, the higher the mortality rate. In addition, post-reproductive mortality is lower for women who had a husband who died after 1994. Mortality is higher among women who experienced the death of one or more of their children during infancy. Finally, and in line with our expectation, there is a strong decrease in mortality across birth cohorts. Per 10-year birth cohort, the mortality rate drops by 13%. Additional analyses that examine whether this trend is linear or curvilinear (results not shown) show that this is a linear trend across all cohorts.

**Table 2:** Cox regression estimates (*e* ^B^) and 95% confidence intervals for survival models explaining post-reproductive mortality among ever-married women with children born between 1850 and 1910.

	Model 1		Model 2	
	b	CI	b	CI
Social power	0.98	0.96–1.00	0.98	0.96–1.00
# reproductive years married	1.00	1.00–1.01	1.00	1.00–1.01
Age difference husband and wife	1.01 ^a^	1.00–1.01	1.01 ^a^	1.00–1.01
Experienced infant death	1.13 ^c^	1.06–1.21	1.13 ^c^	1.06–1.21
Widowed	1.00	0.94–1.07	1.00	0.94–1.07
Husband died after 1994	0.42 ^b^	0.24–0.74	0.42 ^b^	0.24–0.74
Year of birth (per 10 year)	0.87 ^c^	0.86–0.89	0.81 ^c^	0.77–0.85
Two children (refcat = one child)	0.89 ^a^	0.80–0.97	0.84 ^b^	0.76–0.93
Three or four children	0.97	0.88–1.06	0.93	0.85–1.02
Five or more children	0.98	0.90–1.08	0.94	0.86–1.04
Two children * year of birth			1.11 ^b^	1.03–1.19
Three or four children * year of birth			1.11 ^b^	1.04–1.19
Five or more children * year of birth			1.07 ^a^	1.01–1.14
N	5,474		5,474	
df	10		13	
-2 log likelihood	82,252.2		82,240.9	

^a^ *p* < 0.05.

^b^ *p* < 0.01.

^c^ *p* < 0.001.

The results for number of children in Model 1 of Table 2 show that the mortality rate for women with two children is 11% lower than for women with one child. This difference is statistically significant (p = .013). Mortality rates for women with three children or more do not differ from those of women with one child. In additional analyses (results not shown), we tested whether the effects for number of children were proportional. No violations of the proportionality assumption underlying Cox’s regression model were found, so the difference between women with two children and women with other parities are stable across the whole age range from 50 years of age onwards. Thus, for all birth cohorts together we do not observe a linear trade-off between women’s fertility and their post-reproductive longevity.

**Fig 4:**
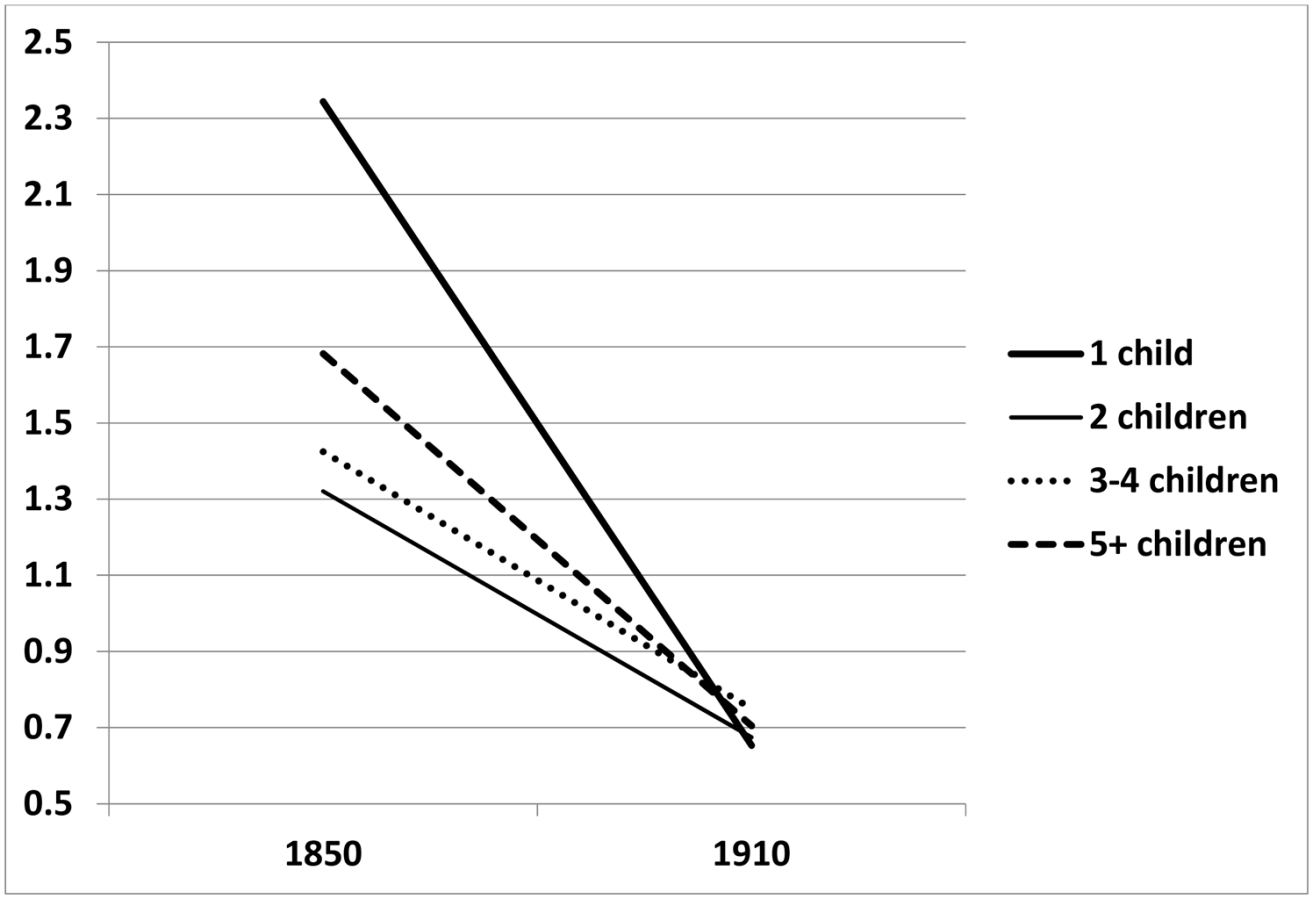
Trends in mortality rate by period across cohorts, based on Model 2 in Table 2.

Model 2 tests our hypothesis that the trade-off between fertility and post-reproductive longevity diminished during the epidemiological transition. In this model, we added linear interaction terms between birth year and number of children, to examine whether the effect of number of children changes across birth cohorts. Adding these interaction terms improved the model fit (*Χ* ^*2*^ = 11.25, df = 3, *p* = .01). First, we notice that no significant changes occur in any of our control variables between Models 1 and 2. With regard to the effects of parity, we observe statistically significant effects for the interaction between birth cohort and having two, three or four, or five or more children. To facilitate the interpretation of these effects, we plot the change in relative mortality rates for all parity categories across cohorts. The results are presented in Fig 4. In this figure, the mortality rates for women with one child born in 1890 are arbitrarily fixed to 1. Among the oldest cohorts in the sample, those born in the 1850’s, mortality rates are markedly different dependent on the fertility history. Women with two children have the lowest mortality rates, followed by women with three or four children–having a mortality rate that is 7% higher–and women with five children or more–having a mortality rate that is 27% higher than ever-married women with two children. Women who had just one child have by far the highest mortality rates, being about 78% higher than those of women with two children. Across cohorts, the differences in post-reproductive mortality rates diminish and among cohorts born around 1910, these differences have completely vanished. In general, this pattern of effects shows that a U-shaped association between parity and post-reproductive longevity in the earlier cohorts diminishes over time.

Finally, we also tested whether the effect of parity differs by social class by including interaction terms between social class and each of the parity categories. This does not improve the fit of this model (*Χ* ^*2*^ = 0.25, df = 3, *p* = .97). None of these interaction terms reaches statistical significance. Thus, the effect of parity on post-reproductive survival of ever-married women with children does not vary by social class.

## Discussion

Several hypotheses have been put forward to explain the mixed results of studies on the relationship between women’s fertility and their post-reproductive longevity. In this study, we focused on the disposable soma theory. A negative relationship between women’s fertility and their post-reproductive longevity can be understood as an evolutionary trade-off between reproduction and survival. This trade-off could be mediated by maternal depletion of (metabolic) resources or by genes selected for fertility but with pleiotropic effects later in life such as the immune system. We studied the relationship between women’s fertility and their post-reproductive longevity during the epidemiological transition in the Netherlands, a period for which it is expected that the evolutionary trade-off between women’s fertility and their survival will fade as environmental hazards considerably declined. We rigorously controlled for socio-economic differences using estimators of social power and early family mortality to distinguish the assumed trade-off from direct environment-driven associations.

When all female birth cohorts were taken together, we did not find a trade-off between women’s fertility and their post-reproductive longevity. However, the relationship between women’s fertility and their post-reproductive longevity markedly changed during the epidemiological transition. Around 1850, before the transition had started, we found a U-shaped relation between parity and post-reproductive longevity: the least and most parous women had a higher risk of mortality than women with parity between three and five. Among parous women, the relationship between fertility and longevity completely faded in a period of two to three generations, when mortality from infectious diseases decreased to a minimum.

The fading relationship between fertility and longevity for women gives some support to an evolutionary trade-off between fertility and longevity. We found a U-shaped association between parity and post-reproductive survival in the earlier cohorts, that were under higher environmental pressure. This association disappeared in later cohorts. We found the interaction between parity and time in all social classes in our sample, suggesting that differences in parental resources or socio-economic pressures are not relevant. This makes it less likely that the differences can be attributed to differences in socio-economic pressures or general hardship, as the maternal depletion hypothesis would suggest. We cannot fully rule out maternal depletion as an explanation for the disappearance of the negative relationship between women’s fertility and longevity. Together with the drop in mortality from infectious diseases, the GDP per capita rapidly increased [33]. It has been argued that a maternal depletion effect will be more manifest when resources are scarce [8, 10, 34]. At the same time, we have tried to overcome this by controlling for early child mortality in the family and bereavement.

The higher mortality of women with one child in the earlier cohorts contradicts the disposable soma theory, which would predict a linear negative relationship between parity and post-reproductive survival. In the period under study, having only one child was exceptional in The Netherlands. The U-shaped association is in line with the more reliable historic studies that Gagnon [2] reviewed. It is plausible that unmeasured health problems explain both the lower fertility and the higher mortality of the mothers. Another possibility is that the timing of childbirths explains more of post-reproductive survival than the total fertility does: women who reproduce longer both have more children and have a higher post-reproductive life expectancy [2].

We were able to cover a crucial period of over half a century with complete life histories across The Netherlands by using the Historical Sample of the Netherlands. To further disentangle the various mechanisms behind the relationship between women’s fertility and longevity, there is a need for research which makes use of data from varying epidemiological, cultural and economic contexts. Appropriate models should include information about the genetic profile of the women, the development of their health and their socio-economic status over time, the social and material support from their children and the women’s cause of death. Most historical datasets will lack such detailed information. However, these data may be collected from contemporary societies that still live under largely preindustrial conditions.
